# On the emergence of atypical *Vibrio cholerae* O1 El Tor & cholera epidemic

**Published:** 2011-04

**Authors:** Ana Carolina Paulo Vicente

**Affiliations:** Oswaldo Cruz Institute, FIOCRUZ Rio de Janeiro, Brazil anapaulo@ioc.fiocruz.br

*Vibrio cholerae* shows a remarkable variability easily seen by the identification of over 200 O serogroups in the species. However, only some lineages, of the O1 and O139 serogroups, have been able, so far, to trigger epidemic and pandemic cholera. The clinical O1 strains were further classified, on the basis of phenotypic and genotypic markers, into two biotypes: classical, which had driven, at least, the sixty cholera pandemic, and El Tor, the aetiological agent of the current cholera pandemic (7^th^ cholera pandemic). The El Tor strains were first isolated on the Indonesian island of Sulawesi in 1961 and after that outbreaks, due to this lineage, started occurring in Bangladesh, India and Russia. Only more than a decade from its beginning the 7^th^ cholera pandemic reached the North of Africa continent^1^.

By the end of the last century, two events changed centenary cholera concepts: the emergence of *V. cholerae* O139 serogroup^2^, causing epidemic cholera, and the discovery that the cholera toxin genes, which represent the major virulence factor in the cholera disease, are carried on the genome of a filamentous phage, CTXPhi^3^. The virulence, in the cholera syndrom, is multi-factorial, however, cholera toxin plays a key role in the severe secretory diarrhoea, that characterizes the devastating outbreaks. As being part of a phage genome the cholera toxin genes have an enourmous possibility of mobility by lateral gene transfer mechanism. In a short period of time it was determined that O139 serogroup was, in fact, an El Tor strain in a new robe^4^. This new *V. cholerae* phenotype was the consequence of changes in the O antigen-coding region but the overall traits and core genetic background of the El Tor biotype were maintained^5^. The expectation that this new serogroup would replace the major lineage driving the seventh cholera pandemic and would trigger the eighth cholera pandemic was not confirmed and, so far, *V. cholerae* O139 serogroup has been causing restricted outbreaks in the Indian subcontinent.

Cholera and *V. cholerae* continue challenge to the world and by the beginning of the 21^st^ century a new scenario emerged when variants of typical *V. cholerae* El Tor biotype were identified, causing cholera outbreaks, in many Asian and African countries^5^–^7^. Interestingly, these variants harbour some phenotypic and genotypic traits that are characteristics of the two major pandemic *V. cholerae* biotypes. Classical and El Tor biotypes were successful in determining cholera pandemic during the last century, but the emerging atypical El Tor strains are likely to have a similar performance to these biotypes considering the epidemiology of these variants. Moreover, in some of the outbreaks, due to these atypical El Tor strains, a much higher proportion of patients presenting severe dehydration have been noticed. This is in contrast with what usually occurs when typical El Tor strains are in charge. In general, cholera produced by the classical biotype is more severe than that caused by El Tor, on the other hand, El Tor biotype presents an higher ecological fitness, with long persistence in the aquatic environment^8^. In the Fig. the current geographic distribution of these variants is represented. The black labelled countries are those where distinct atypical El Tor strains, the Matlab and Mozambique variants and the altered and hybrid El Tor, were characterized as determinants of outbreaks and, in some of these, there is no longer cholera outbreak due to typical El Tor lineage^5^. The hatched countries are those where one or more of the variants have been associated with cholera cases.

**Fig.**
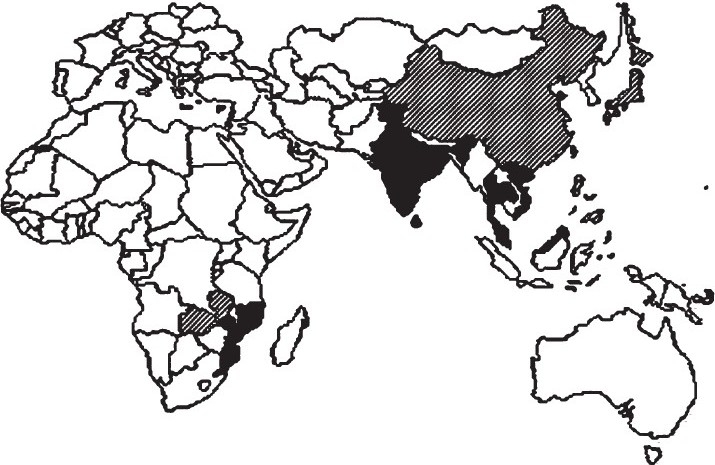
Map showing the scenario of the spread of atypical *V. cholerae*. Countries where any atypical strain was the determinant of cholera outbreak (black label 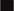) and countries that have already isolated atypical strains from cholera cases (hatched label 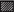).

Na-Ubol *et al*^9^, in this issue, enlarge this picture presenting a detailed genetic characterization of one representative collection of clinical *V. cholerae* O1 from Thailand recovered over 24 years (1986-2009). Their results showed the hybrid biotype of *V. cholerae* O1 strains co-circulating in Thailand, with the typical El Tor, since 1986. Considering their collection, the typical El Tor strains were found for the last time in 1992, when the El Tor variant biotype carrying ctxB ^C^/rstR^E/C^ emerged in the country. During 1993-2009 the majority of the strains characterized belonged to El Tor variants group including the hybrid biotype that harbours the classical cholera toxin allele ctxB. Therefore, Thailand is one of the Asian countries where the typical El Tor strains were displaced by the variants.

All the evidences on the shifting in the prevalence of the major *V. cholerae* El Tor lineage, as determinant of cholera epidemic, stress the urgency to perform genetic characterization of clinical *V. cholerae* isolates from the ongoing, as well previous cholera outbreaks.

The current scenario of the distribution in the world, of the atypical El Tor strains causing cholera epidemics, reveals that distinct pathogenic strains harbouring a combination of biotype traits, that can be genes coding for both virulence and ecological fitness, are likely to be successful as important public health threats.
